# Conduct of a personal radiofrequency electromagnetic field measurement study: proposed study protocol

**DOI:** 10.1186/1476-069X-9-23

**Published:** 2010-05-20

**Authors:** Martin Röösli, Patrizia Frei, John Bolte, Georg Neubauer, Elisabeth Cardis, Maria Feychting, Peter Gajsek, Sabine Heinrich, Wout Joseph, Simon Mann, Luc Martens, Evelyn Mohler, Roger C Parslow, Aslak Harbo Poulsen, Katja Radon, Joachim Schüz, György Thuroczy, Jean-François Viel, Martine Vrijheid

**Affiliations:** 1 Department of Epidemiology and Public Health, Swiss Tropical and Public Health Institute, Socinstrasse 57, Basel, 4051, Switzerland; 2 University of Basel, Petersplatz 1, Basel, Switzerland; 3 Laboratory for Radiation Research, National Institute for Public Health and the Environment (RIVM), Bilthoven, 3720 BA, the Netherlands; 4 Safety & Security Department, Austrian Institute of Technology GmbH, Seibersdorf, 2444, Austria; 5 Centre for Research in Environmental Epidemiology (CREAL), Municipal Institute of Medical Research (IMIM), Barcelona Biomedical Research Park (PRBB), Doctor Aiguader, 88, Barcelona, 08003, Spain; 6 Institute of Environmental Medicine, Karolinska Institute, Stockholm, Sweden; 7 Institute of Non-ionizing Radiation (INIS), Pohorskega bataljona 215, Ljubljajna, 1000, Slovenia; 8 Institute and Outpatient Clinic for Occupational, Social and Environmental Medicine, Hospital of the Ludwig-Maximilians-Universität, Ziemssenstr. 1, Munich, 80335, Germany; 9 Department of Information Technology, Ghent University/IBBT Gaston Crommenlaan 8, B-9050 Ghent, Belgium; 10 Centre for Radiation, Chemical and Environmental Hazards. Health Protection Agency, Didcot, UK; 11 Centre for Epidemiology and Biostatistics, Leeds Institute of Genetics, Health and Therapeutics (LIGHT), University of Leeds, Clarendon Way, Leeds, LS2 9JT, UK; 12 Institute of Cancer Epidemiology, Danish Cancer Society, Strandboulevarden 49, Copenhagen, 2100, Denmark; 13 Department of Non-ionising Radiation, National "Fréderic Joliot-Curie" Research Institute for Radiobiology and Radiohygiene, Anna. str.5, Budapest, 1221, Hungary; 14 French National Institute for Industrial Environment and Risks (INERIS), Parc ALATA Bp2, Verneuil en Halatte, 60550, France; 15 Laboratoire Chrono-Environment (UMR N° 6249), Centre National de la Recherche Scientifique (CNRS), Faculty of Medicine, 2, place Saint Jacques, Besançon, 25030, France

## Abstract

**Background:**

The development of new wireless communication technologies that emit radio frequency electromagnetic fields (RF-EMF) is ongoing, but little is known about the RF-EMF exposure distribution in the general population. Previous attempts to measure personal exposure to RF-EMF have used different measurement protocols and analysis methods making comparisons between exposure situations across different study populations very difficult. As a result, observed differences in exposure levels between study populations may not reflect real exposure differences but may be in part, or wholly due to methodological differences.

**Methods:**

The aim of this paper is to develop a study protocol for future personal RF-EMF exposure studies based on experience drawn from previous research. Using the current knowledge base, we propose procedures for the measurement of personal exposure to RF-EMF, data collection, data management and analysis, and methods for the selection and instruction of study participants.

**Results:**

We have identified two basic types of personal RF-EMF measurement studies: population surveys and microenvironmental measurements. In the case of a population survey, the unit of observation is the individual and a randomly selected representative sample of the population is needed to obtain reliable results. For microenvironmental measurements, study participants are selected in order to represent typical behaviours in different microenvironments. These two study types require different methods and procedures.

**Conclusion:**

Applying our proposed common core procedures in future personal measurement studies will allow direct comparisons of personal RF-EMF exposures in different populations and study areas.

## Background

There has been a substantial increase in environmental exposure to radio frequency electromagnetic fields (RF-EMF) over the last few decades due to the introduction of new technologies, especially those related to wireless communication [1]. This development has led to concerns regarding possible effects of exposure to environmental RF-EMF on health [2-4].

Until now RF-EMF risk assessment has been hampered by the lack of reliable exposure assessment methods. Day-to-day exposure to RF-EMF comes from many different sources producing large variability in small-scale spatial and temporal exposure patterns. Prior to the availability of personal measurement devices, measurement of RF-EMF was complex and time consuming. In particular, concurrent measurements of different RF-EMF sources in many locations, long term measurements and measurements when moving were very challenging. As a result, previous measurement studies have focussed mainly on maximum exposure levels occurring over space and/or time, as appropriate for assessing compliance with safety limits, but not on exposure patterns in the general population such as average personal exposure, time spent above a threshold or rate of change. These quantities are of more interest for health risk assessments and for epidemiological studies. Thus, information about the total RF-EMF exposure of individuals in different populations is scarce. Only crude methods have been used for exposure assessment in epidemiological studies such as self-reported use of mobile phones [5,6], spot measurements of specific sources [7,8], or distances between residential addresses and the nearest transmitter. Distance was shown to be a modest RF-EMF exposure proxy with respect to broadcast transmitters but was inaccurate for mobile phone base stations [1,9-13].

**Figure 1:**
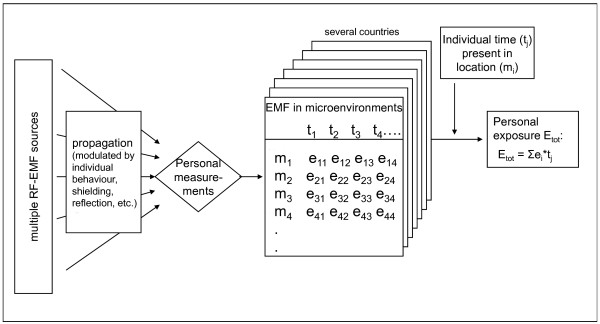
**Personal exposure**. The relation between emissions from RF-EMF, personal measurements, exposure levels (e_ij_) in different microenvironments (m_i_), time spent at different microenvironments (t_j_) and personal exposure (E_tot_).

Personal exposure to RF-EMF depends on exposure levels in the environment and on individual behaviour such as use of wireless communication devices (e.g. W-LAN, mobile or cordless phones) and time spent in different microenvironments (Figure 1). For the purpose of estimating exposure, a microenvironment is considered a spatial compartment where an individual spends a certain period of time and exposure can be characterized during that time period. Linkage between behavioural factors and RF-EMF levels in different microenvironments is possible using personal measurements and a time-activity diary. The availability of RF-EMF exposure meters (exposimeters) means that personal RF-EMF exposure to multiple sources in the everyday environment can be more accurately assessed. Several studies have demonstrated the applicability of exposimeter measurements in population samples [13-21]. Comparing exposure levels between countries using data from these first studies is problematic, however, because different types of measurement devices and/or different measurement and analysis procedures have been used. This means that observed differences in exposure measurements may be due to methodological differences and may not reflect real exposure differences between populations. In order to accurately compare exposure levels between or even within countries, it is of crucial importance to conduct comparable measurements. The aim of this paper is to propose basic requirements for the conduct of personal measurement studies based on the current preliminary insights into this topic. This includes descriptions of the study instruments and methodological issues such as selection procedures for study participants, handling of the exposimeter, collection of other relevant data, data handling and reporting of the results.

## Research protocol

### Objectives

Personal measurement studies usually have one of the following two objectives. Firstly, to determine personal exposure distribution in the population of interest (population survey). Secondly, to characterize typical exposure levels in different microenvironments in the area of interest such as public transportation or outdoor urban areas (microenvironmental measurement). These two objectives should be clearly differentiated because they have major implications for the study methods (Table 1).

**Table 1:** Comparison between a population survey and a microenvironmental measurement campaign.

	Population survey	Microenvironmental measurement study
Unit of observation	Individual	microenvironment*

Requirement for the study sample	representative for the population of interest	representative in terms of exposure-relevant behaviours for the population of interest

Selection of participants	random and representative sample needed	convenient sample is sufficient oversampling of rare exposure-relevant behaviours

Motivation of participants	part of the random sample will not be motivated	convenient sample is more motivated on average

diary	basic and simple, if any at all	compulsory

Measurement duration	as long as reasonable for the participants	not crucial

Sample size	many individuals	many measurements from numerous microenvironments of the same type

* For the purpose of estimating exposure, a microenvironment is considered a spatial compartment where an individual spends time and exposure can be characterized during that time.

### Study instruments

#### Personal exposimeters

So far two different types of exposimeters have been applied in exposure measurement studies: the EME SPY 120/121 (SATIMO, France) and the ESM-140 (Maschek, Bad Wörishofen, Germany). The latter device is easier to carry, however, measurements of radio FM and TV bands are not possible (Table 2). The EME SPY has an isotropic antenna whereas the ESM-140 takes into account shielding of the body and its antenna is designed in a way that full isotropy is only achieved when the meter is carried on the upper arm, which is a drawback for measurements during the night. Only the EME SPY is suitable for RF-EMF measurements in a stand-alone position. Another disadvantage of the ESM-140 is that accuracy of the differentiation between up- and downlink measurements in the mobile phone bands is limited [17,22]. Recently, SATIMO developed a new type of personal exposimeter (EME YPY 140) with markedly improved characteristics [23]: i.e. increased frequency range (80 MHz-6 GHz), increased dynamic range (sensitivity: 0.005 to 5 V/m), a more appropriate complex signal assessment, a reduced sampling period (from 330 μs to 18 μs) which is relevant for signals with short pulse duration such as DECT and W-LAN, and a reduction of the device's size by a third (Table 2).

**Table 2:** Overview of exposimeters.

Band	Frequency [MHz]	Description	ESM-140	EME SPY 121	EME SPY 140
FM	88-108	FM radio broadcasting	no	yes	yes

TV3	174-223	TV broadcasting	no	yes	yes

Tetrapol	380-400	Mobile communication system for closed groups	no	yes	yes

TV4/5	470-830	TV broadcasting	no	yes	yes

GSM900 uplink	880-915	Transmission from handset to base station	yes^1^	yes	yes

GSM900 downlink	925-960	Transmission from base station to handset	yes^1^	yes	yes

GSM1800 uplink	1710-1785	Transmission from handset to base station	yes^1^	yes	yes

GSM1800 downlink	1805-1880	Transmission from base station to handset	yes^1^	yes	yes

DECT	1880-1900	Digital enhanced cordless telecommunications	yes^1^	yes	yes

UMTS uplink	1920-1980	Transmission from handset to base station	yes^1^	yes	yes

UMTS downlink	2110-2170	Transmission from base station to handset	yes^1^	yes	yes

W-LAN	2400-2500	Wireless Local Area Network	yes	yes	yes

WIMAX	3400-3800	Worldwide Interoperability for Microwave Access	no	no	yes

WI-FI	5150-5850	A wireless IEEE 802.11standard	no	no	yes

**Other characteristics**:					

Measurement range (V/m)			0.01-70	0.05 - 10	0.005-5

Measurement cycle			0.5 - 10s	4 - 255s	4 - 255s

Storage capacity (number of measurements)			260,000	12,540	80,000²

Size (L × W × H in mm)			115x45x29	193 × 96 × 70	169 × 79 × 46

Weight (in g)			87	450	400

Marker (to register events)			yes	yes	yes

Overview of the exposimeters (ESM-140, EME SPY121) that were previously used in studies and the newly developed EME SPY 140 [23].

^1^combined bands for the frequency range between 880 and 960 MHz and for 1700-2200 MHz.

^2^Theoretical capacity taking into account the capacity of the memory component and the number of bytes to save for measurement, but may not be achieved due to battery life.

Currently, this new device seems to be most appropriate for future measurement studies. Nevertheless, the performance of other exposimeters that may be developed in the future should be thoroughly evaluated as well. Basic requirements for an eligible device are the measurement accuracy, an optimal isotropy, the ability to differentiate between different frequency bands (in particular between up- and downlink in the mobile phone bands) and to be acceptable to study participants. The latter is particularly important for population surveys, as study participants have to agree to carry such a device over a relatively long time period in order to obtain robust measurements of their typical exposure.

#### Geographic Position System (GPS) device

In addition to the exposimeter, the use of a GPS device that geo-locates the personal RF-EMF measurements is a useful adjunct to exposure studies. This procedure has been successfully applied in the Netherlands [24] and in Belgium [25]. Ideally, such a GPS device should be directly implemented in future exposimeters but has not been so far.

The measured electric field strength can be plotted on a Google Earth map at the latitude, longitude position of the coordinates from the GPS logger. This visualization can be done for all frequency bands and can be used as a quality control tool to check the plausibility of the entries in the time-activity diary (see below). The GPS and exposure data can also be applied as input data for the development of physical or empirical propagation models [26] including spatial characteristics. Also the exposure can be coupled to data from mobile phone network providers for spatial correlation between exposure and the network layout.

#### Time-activity diary and questionnaire

In order to obtain interpretable measurements, study participants have to fill in a time-activity diary. The diary will provide additional information about the type of microenvironment experienced by the participants when using the measurement device. The diary needs to be simple and easily comprehensible but also provide standardised information which can be used for data analysis. Thus, there is a certain limitation to what can be achieved in terms of spatial and temporal resolution. In particular, one has to be aware that less demanding tasks can be required from the study participants in a random population sample compared to a convenience sample, which is generally more motivated. For a convenience sample or for hired participants, we propose, as a minimal requirement, that the following microenvironments are considered in all future microenvironmental measurement studies in order to obtain comparable measurements between countries: at home (bed room, living room, other rooms, outside at home [e.g. balcony, garden]), at work, being outdoors (not at home), shopping, driving a car, travelling by public transportation, riding a bicycle, being at bus/train stations, school/universities/courses, or at any other places. If necessary, in a specific geographic context or study objective, the diary may be complemented with additional details of microenvironments. Instead of a paper and pencil diary, use of hand-held computers (personal organizers) may be an option but this has not been done so far in this context and should be piloted.

It is important to note that personal exposimeter measurements do not differentiate between exposure from the participants' equipment (e.g. mobile phone) and other sources for which the distance to the exposimeter may be the same. Such a differentiation is needed from the exposure perspective because the participants' equipment radiates closer to the body causing much more absorption of radiation. Information about participants' own use of equipment that emits RF-EMF should be collected. For exposure to RF-EMF from mobile phones, the best method is to obtain traffic data of the mobile phone from the network operators of study participants during the measurement period. This would still need to be linked to mode of use (hands-free, in-car etc.). Another option is to use information that is stored in the mobile phone. This is not possible for all phone types, however, and generally not for cordless phones. The third option is that study participants note all calls in the time-activity diary or press an event marker of the exposimeter (only available for ESM-140). However, in this case some of the calls may be missed, because the participants forget to note them. Such missing data may not be randomly distributed between study participants or microenvironments and therefore may introduce bias to a study.

In addition to the diary, a questionnaire about other exposure-relevant items and general exposure-relevant behaviour during the measurement period is useful for interpreting the data. Exposure-relevant behaviour relates to the typical use of mobile and cordless phones, use of a hands-free device and the physical location where the phone is kept when such a device is used, use of wireless networks, possible occupational exposures as well as socio-economic variables, housing characteristics and factors that might indicate exposure avoidance behaviour (e.g. concerns about adverse health consequences from electromagnetic fields).

### Study procedures

#### Measurement duration

Detailed study procedures depend on the specific aims of a study. Measurement duration for a participant should be at least 24 hours and not exceed 1 week. Short measurement periods may not be representative of the behaviour of the participants (e.g. weekend vs. workday behaviour). Long measurement periods, on the other hand, may result in a decreased diary quality due to participation fatigue. This may be particularly the case in a random population sample because a part of the sample may not be motivated for study participation. Shorter measurement periods make the logistics of exchanging devices more complex. Due to the limited number of measured values that can be stored (e.g. 7168 for the EME SPY 120), the sampling interval is determined by the duration of the measurement period. The optimum sampling interval should be as short as possible and is determined by the duration of the measurement period and the storage capacity of the device. It should also be constant within a given study to facilitate internal comparison. In conclusion, the choice of the measurement duration is not crucial and should be based on logistic and methodological considerations. As a rule, in population surveys, the exposure of interest is that of the individual and the measurement period should be as long as reasonable for participants whereas for microenvironmental measurement studies, a high number of measurements per microenvironment can be obtained with a high sampling rate within a relatively short measurement period.

#### Selection of study participants

In the case of a population survey, the unit of observation is the individual and a randomly selected representative sample of the population is needed to obtain reliable results. For microenvironmental measurements, study participants should be selected in order to represent typical behaviours in different microenvironments.

Participants in a population survey would ideally be selected from population registries. With other recruitment approaches care must be taken to avoid exposure-related selection bias. For instance, people using mobile phones exclusively may be underrepresented in the telephone directory, resulting in an underestimation of mobile phone use in a cohort selected in this way. For population surveys, participation bias is of concern and thus incentives may help to obtain a high participation rate. We also strongly recommend a two-tier recruitment process. First, a short questionnaire should be distributed to the target population with exposure-relevant items including a question as to whether participation in the measurement study is agreed. This needs little effort and the return rate will be probably high. The data can then be used to evaluate how representative the study participants are of the rest of the population in terms of exposure-relevant behaviours and socioeconomic factors.

The sample size that is needed for such a population survey is still difficult to define with the current limited knowledge about the exposure variability in the various populations. Because exposure-relevant behaviour is expected to be related to age, gender, type of residential area (urban, suburban, rural), and time (workday vs. weekend/holidays; day vs. night), we recommend that study participants are selected from predefined strata, thus applying a stratified random sampling. In order to ensure comparability between studies we advise to use the following age groups for analyses: primary school children (depending on the country, about 7-12 years), secondary school children and adolescents (about 13-19 years), young adults (20-35 years), adults (35 up to retirement), and retired people. In total, such a classification results in 30 different strata by age, gender and type of residential area. Future studies may decide not to consider all strata, but if such studies use these predefined strata for selection of study participants and reporting of the measurement results, comparability between studies will be enhanced and exposure differences due to different study sample compositions will not be wrongly attributed to differences between study areas. In order to obtain representative results for the population of interest the following potentially exposure-relevant characteristics or factors should be representatively distributed in each stratum: socioeconomic status, use of wireless communication devices, use of public transport, and day of week. In summary, directly determining population exposure from exposimeter measurements is resource intensive and requires a large study size because the unit of observation is an individual.

Study participant selection criteria are different for microenvironmental measurement studies because the unit of observation is clearly delineated, such as train or outdoor urban residential area. This does not require a random sample but rather study participants who represent the whole range of exposure-relevant behaviours and activities in the area of interest. For instance, children, adolescents and adults behave differently in their daily life and spend their time in different microenvironments (e.g. school vs. workplace). Thus, it is advised to select a few participants from each of the above mentioned strata. A convenience sample of motivated people will help to ensure high compliance with the study protocol. One could even consider hiring study participants who take measurements in predefined microenvironments following a predefined protocol, as in a Dutch measurement study [24]. Because exposimeters can store several thousands of measurements, it is relatively simple to obtain a large amount of measurements per microenvironment. Nevertheless, in order to represent the full range of exposure distributions and behavioural aspects in each type of microenvironment, measurements from numerous participants should be collected for each type of microenvironment. For instance, it is important to have measurements from many different railway stations to obtain a reliable estimate of the general exposure situation in railway stations in a study area.

#### Instructions for the study participants

Handling of the exposimeters affects the measurements and thus the same procedures have to be applied to obtain comparable results from several studies. Ideally, exchange of the measurement devices should take place at the home of the study participants, which would also offer the opportunity for the researcher to take additional objective data about the exposure situation (e.g. spot measurements or data on housing characteristics). Alternatively, participants may collect the instrument at the study centre. In this case one has to be aware that the measurement day does not reflect the typical activity of the study participants, and this should be considered when determining the individual exposure level. In a microenvironmental measurement study one has to ensure that measurements from a specific microenvironment in the vicinity of the study centre are not over-represented in the final data set as this microenvironment may not be representative for all microenvironments of the same type.

An important aspect of obtaining accurate and replicable measurements is the placement of the exposimeter. Based on previous experience we propose that the participants carry the exposimeter in a camera bag in order to keep the position of the device stable. Mobile phones should not be placed in the same bag. A camera bag is impractical when sitting down, and thus in this case the device should be placed in the vicinity of the person. This also minimizes shielding effects by the person which are of concern when the exposimeter is placed on the body [27]. Thereby the participants should be advised not to place the exposimeter at exactly the same place each time and to move it a little bit at least every hour (except during the night) in order to obtain more representative values. When changing the room, participants should carry the exposimeter with them. The exposimeter must not be placed on the floor, on a window sill or in the close vicinity (less than 30 cm) of a wall or of an electrical device.

#### Maintenance and calibration of the exposimeters

**Figure 2:**
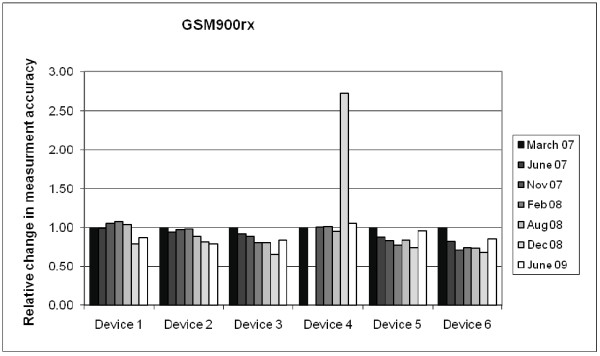
**Functional test of exposimeters**. Example of functional tests of the GSM900 downlink band of the six devices used in a Swiss study (QUALIFEX) conducted by the Federal Office of Metrology in Wabern, Switzerland. All relative changes refer to the V/m units. The tests revealed a problem with device number 4 in December 2008. All other changes were within the measurement uncertainty of ±2 dB.

Although exposimeters are calibrated by the manufacturer prior to delivery, it is imperative to conduct further functional tests and calibrations with each device during conduct of the research. Calibration factors may drift with time. Devices may also break down or become corrupt during the course of the study, as participants have to carry them around all the time, presumably resulting in some rough handling. Functional tests should reveal crude deviances from proper functioning and any time shift in the measurement accuracy. Simple functional tests are recommended each time before the device is distributed to a new participant. Basic requirements for functional tests are replicable exposure situations with a transmitter for each frequency band. The absolute measured values of the functional tests are less important than the relative changes between the tests (see Figure 2). A major change in the measurement reading (e.g. >3 dB) indicates that the device may no longer be functioning properly and calls for a thorough investigation, any repairs necessary and re-calibration of the device. Calibration is an extensive procedure because realistic signals have to be used in each frequency band. Continuous wave signals will not produce correct results for pulsed signals [28]. Moreover, isotropy and linearity have to be considered when performing a calibration. There is also a need to take into account that strong signals outside the frequency bands measured by the exposimeters might couple into them or that coupling between adjacent different bands of the exposimeters can occur, e.g. between GSM 1800 downlink and DECT (Table 2). Frequency specific calibration factors have to be determined, i.e. calibration factors might differ within the same frequency band depending on the carrier frequency. This means that different calibration factors can be observed, e.g. at 90 or 100 MHz in the FM band [28]. As a consequence, the calibration factor for each frequency band should take into account the average distribution of the EMF within that band in the study area.

#### Data Management and Cleaning

Data management includes combining exposimeter data with diary and GIS data. The temporal measurement resolution is usually much denser than the diary resolution and thus cleaning of the dataset is required. Any obvious discrepancies between measurements and diary or GPS data should be resolved. The geo-referenced data allow easy detection of a change of place of the exposimeter. Moreover, the measurement pattern of various frequency bands usually changes abruptly when moving, e.g. going inside from outside or vice versa. Thus, the data should be visually inspected and the plausibility of the diary entries should be checked. Based on experience from previous studies, the most common problem is a time shift between measurements and diary entries, which should be corrected in the diary. An obvious change of the location in the measurement file without a corresponding entry in the diary indicates that a relevant entry in the activity diary has been forgotten. Conversely, a recorded change of location in the diary without a corresponding change in the measurement file indicates that the exposimeter has not been carried on the person. Such measurements or diary entries should be adapted in the most plausible way or removed from the data. All changes should be flagged in the data sheet for later sensitivity analyses. In general, a conservative data cleaning approach is recommended i.e. to change as little as possible. Previous experience suggest that such changes do not have a major impact on the summary statistics of the measurements [15]. Nevertheless, a computerized procedure instead of visual inspection is considered to be more objective and not to introduce systematic differences between studies. Such a procedure has not been developed so far but a common computerized procedure would enhance the reproducibility and reliability of cross-study comparisons.

#### Data analysis

The main challenge for data analysis is measurements below the detection limit. Although the detection limit is expected to be reduced in future exposimeters, adequate statistical methods must be used to account for sub-threshold measurements [29]. For summary statistics we recommend the use of robust regression on order statistics (ROS), which has been shown to produce reliable summary estimates of personal measurements with a substantive proportion of nondetects [30]. Nevertheless, some caution is warranted if only a few and similar values are recorded above the detection limit. In this case, the estimated data distribution produced by ROS is unreliable. In addition to ROS, a variety of statistical methods for censored data are implemented in the package NADA for the R statistical software [31]. We also recommend regression modelling methods that allow nondetects and non-parametric score tests for censored data, such as the Peto-Peto test.

The presentation of the results depends on the aim of the study: in a population survey the focus is on the distribution of the individual exposure in the study population. This can also be done for microenvironmental studies as shown in Table 3; however, lack of representativeness of the study sample has to be taken into account when interpreting the data. Data distribution includes the average level as well as other exposure metrics potentially relevant for health such as time spent above a certain threshold, rate of change, or other measures reflecting the intrinsic structure of the exposure as done in [32]. Such an analysis does not necessarily require diary data and can also consider different time periods separately such as weekend vs. workday; daytime vs. night (Table 3). In addition, factors that affect individual exposure (e.g. age, gender and use of communication devices) should be analyzed in a population survey using regression modelling. In general, population surveys will be limited for comparing different microenvironments because the diary will provide less detailed information about the activity compared to a microenvironmental measurement study. However, they will directly inform about the distribution of RF-EMF exposure in the population of interest.

**Table 3:** Distribution of total (all sources) individual exposure at different places and times in a Swiss study sample (partly reprinted from [15]).

	Arithm. mean	Minimum	5% quantile	25% quantile	Median	75% quantile	95% quantile	Maximum
**Average (mW/m**^**2**^**)**	0.134	0.014	0.030	0.054	0.092	0.163	0.351	0.881

- Daytime	0.164	0.014	0.034	0.070	0.127	0.209	0.445	1.063

- Nighttime	0.076	0.003	0.005	0.014	0.028	0.086	0.245	1.367

- Workday	0.134	0.013	0.027	0.055	0.096	0.170	0.353	0.776

- Weekend	0.133	0.007	0.014	0.031	0.064	0.148	0.474	1.243

**Time above 1 V/m (%)**	0.453	0.016	0.046	0.134	0.255	0.509	1.201	8.442

- Daytime	0.629	0.000	0.038	0.174	0.359	0.697	1.988	8.754

- Nighttime	0.083	0.000	0.000	0.000	0.000	0.072	0.313	2.101

- Workday	0.447	0.000	0.036	0.127	0.254	0.500	1.409	5.836

- Weekend	0.458	0.000	0.000	0.052	0.157	0.365	1.714	14.958

**Rate of change (mW/m**^**2**^**)^1^**	0.128	0.011	0.025	0.060	0.102	0.172	0.299	0.484

- Daytime	0.181	0.004	0.018	0.062	0.170	0.260	0.430	0.590

- Nighttime	0.037	0.000	0.000	0.002	0.006	0.021	0.237	0.351

- Workday	0.133	0.003	0.018	0.048	0.117	0.191	0.328	0.480

- Weekend	0.117	0.000	0.004	0.018	0.054	0.189	0.413	0.812

^1^; m = measurement

In a microenvironmental measurement study the focus of the analysis is the exposure distribution in different microenvironments. Thus, the data for each microenvironment from all study participants can be pooled and subsequently summary statistics can be calculated using robust regression on order statistics. In addition, mean exposure contributions of different RF-EMF sources may be presented to evaluate the importance of different sources in various microenvironments. Such an example is given in Table 4. Note, however, that measurements from the same individual are clustered. Thus, for statistical testing of differences between microenvironments multilevel regression modelling (random effect models) are needed, although, to our knowledge, no such method for censored data is implemented in standard statistical software so far.

**Table 4:** RF measurement mean values for different frequency bands (V/m) according to regression order statistics method (Besançon and Lyon, France, 2005-2006, 377 participants) (reprinted from [21]).

	n° of measurements	FM	Tetrapol	TV 4&5	GSM Tx	GSM Rx	DCS Tx	DCS Rx	DECT	UMTS Tx	UMTS Rx	WiFi	Total field
**Total**	2,493,211	0.044	0.005	0.016	0.013	0.018	0.012	0.015	0.037	0.036	0.037	0.038	0.201

**Area**													
Besançon	1,221,716	0.052	0.001	0.016	0.011	0.014	0.006	0.011	0.032	0.045	0.050	0.052	0.201
Lyon	1,271,495	0.036	0.008	0.016	0.016	0.022	0.018	0.020	0.041	0.020	0.034	0.020	0.202

**Place of residence**													
urban	625,140	0.071	0.002	0.019	0.010	0.028	0.017	0.025	0.038	0.044	0.031	0.046	0.231
periurban	1,272,213	0.039	0.008	0.015	0.014	0.016	0.011	0.014	0.038	0.038	0.040	0.037	0.201
rural	595,858	0.013	0.005	0.012	0.015	0.009	0.010	0.006	0.034	0.019	0.050	0.042	0.156

**Time period**													
day	1,657,991	0.044	0.004	0.014	0.017	0.018	0.013	0.017	0.037	0.030	0.036	0.036	0.204
night	835,220	0.045	0.040	0.026	0.006	0.018	0.010	0.012	0.037	0.050	0.043	0.040	0.197

**Age category**													
youths	727,878	0.039	0.001	0.015	0.017	0.019	0.014	0.014	0.035	0.040	0.033	0.028	0.188
adults	1,765,333	0.047	0.007	0.016	0.012	0.018	0.011	0.016	0.038	0.037	0.039	0.042	0.206

**Microenvironment**													
home	1,577,162	0.045	0.008	0.022	0.010	0.017	0.010	0.012	0.041	0.044	0.044	0.037	0.200
workplace	543,868	0.047	0.005	0.014	0.014	0.017	0.014	0.021	0.030	0.025	0.040	0.043	0.205
transportation	187,699	0.044	0.005	0.012	0.030	0.027	0.024	0.024	0.025	0.027	0.033	0.040	0.215
walk	37,706	0.062	0.007	0.012	0.020	0.035	0.022	0.035	0.032	0.030	0.028	0.042	0.233
bicycle, motorcycle	8,310	0.044	0.023	0.019	0.023	0.035	0.027	0.029	0.026	0.070	0.029	0.040	0.227
car	120,378	0.037	0.005	0.012	0.031	0.026	0.022	0.022	0.024	0.025	0.038	0.039	0.204
bus, tramway	14,390	0.055	0.002	0.017	0.034	0.028	0.040	0.024	0.020	0.004	0.027	0.042	0.238
train,underground	6,915	0.050	0.001	0.011	0.071	0.017	0.034	0.019	0.030	0.084	0.043	0.053	0.257
others	184,482	0.036	0.007	0.008	0.021	0.025	0.018	0.016	0.028	0.012	0.024	0.033	0.192

In microenvironmental measurement studies it is more challenging to obtain the distribution of individual exposure of the study sample, in particular if one is interested in subgroups. Just averaging the values from a small sample size will produce unstable and unreliable results. In this case, regression models are useful to predict RF-EMF exposure for different population strata. We suggest using a regression model to predict RF-EMF exposure of the population strata suggested above (i.e. gender, age groups, type of residential area, workday vs. weekend, three socioeconomic levels, user of mobile and cordless phones and owning a WLAN at home) although it may not be possible to include all these strata in each future study. Table 5 shows an example of such a predictive regression model. In this case, young female adults living in an urban area, owning a W-LAN, mobile and cordless phones are chosen as the reference group. Their predicted exposure is 0.11 mW/m². By multiplying up the relevant model coefficients, predicted exposure for any population stratum is obtained. For instance: middle aged men living in suburban areas are exposed to 0.13 mW/m² (= 0.11 mW/m²*0.93*1.27). In principle, such models can be built for each frequency band separately. Note that personal predictors are not meaningful if study participants are engaged to carry out a set of specific activities such as walking, shopping, taking a train, etc.

**Table 5:** Exposure predictions for different strata.

Variable	Category	n	Coefficient	95%-CI	p-value
*Age*	young adults (20-34 y)	56	reference	-	-
	adults (35-64)	69	0.77	0.59;1.01	0.06
	retired people (>64)	6	0.75	0.39;1.42	0.37

*Gender*	Female	74	reference	-	-
	Male	57	0.93	0.72;1.20	0.58

*Place of residence*	Urban	76	reference	-	-
	Suburban	55	1.27	0.97;1.66	0.08

*Ownership of mobile phone*	Yes	119	reference	-	-
	No	12	0.70	0.44;1.11	0.13

*Ownership of cordless phone*	Yes	79	reference	-	-
	No	52	0.91	0.68;1.21	0.51

*Ownership of W-LAN*	Yes	50	reference	-	-
	No	81	0.95	0.72;1.25	0.72

*Socio economic status*	Low	21	reference	-	-
	Middle	17	0.87	0.54;1.39	0.55
	High	93	1.10	0.77;1.58	0.59

Coefficients of a multiple loglinear regression model using data from a Swiss RF-EMF population survey [15]. This model allows predicting average RF-EMF exposure in different population strata

Intercept of the model: 0.11 mW/m^2 ^(95%-CI: 0.08-0.17) (exposure during the day of a female person aged 20-34 living in an urban environment, owning a mobile phone, a cordless phone and wireless LAN at home, with the lowest socioeconomic status).

To calculate total exposure of a woman with the same characteristics but who does not own a mobile phone, the value has to be multiplied by 0.70 resulting in an exposure of 0.08 mW/m^2^. Note that this is only an example to demonstrate the principle of an exposure prediction model. Lack of significance of coefficients for potentially relevant parameters may indicate that a larger sample size is needed for this type of exposure prediction model.

Seasonality and day of the week may be a relevant predictor for personal RF-EMF, although little evidence for this was found in a Swiss study [15]. Nevertheless, these factors may be of importance in other study areas and should be considered. If relevant, they should be included in the data analysis, as a factor in a regression model.

If data about exposure-relevant behaviour are collected, they should be included in the results (e.g. use of mobile phones, W-LAN, etc.). These data may be useful to explain differences between studies and to estimate exposure differences between populations. In addition, secondary data sources can also be used to estimate population exposure taking into account behavioural aspects of the population of interest such as representative survey data on mobile phone use or time spent in public transport.

## Discussion

Newly developed exposimeters allow convenient measuring of personal exposure from multiple sources of RF-EMF in the everyday environment. However, valid comparisons of measurements between studies can only be made if the same basic methods and procedures have been applied. The aim of this paper is to suggest a few key methodological items that should be considered in future studies to enhance the comparability of the results.

The measurement of personal RF-EMF exposure is still a relatively new area of research. Any procedures suggested in this paper are thus still based on somewhat preliminary insights and may be subject to adaptation taking into account results from future studies. Nevertheless, the authors of this manuscript have practical experience from such personal measurements which form the basis of this presentation of the current state of knowledge for the conduct of personal measurement studies.

We consider it important to clearly differentiate between two objectives that can be achieved by such a study: determination of exposure distribution in a target population (population survey) or measurement of RF-EMF levels in different microenvironments (microenvironmental measurement). Both approaches have their merits and their limitations. A population survey needs a considerable larger sample size than a microenvironmental survey because the unit of observation is an individual. A microenvironmental study allows comparison of the exposure levels between different study areas but does not necessarily reflect population exposure because time spent in different microenvironments may differ between study populations. As an example, studies in France and Switzerland found relatively high RF-EMF levels in trains [15,21] and travelling by train is therefore an exposure-relevant behaviour. Thus, to estimate the importance of this aspect for the RF-EMF exposure of the population, one needs data about the use of trains on the population level. Similarly, exposure of young children, who are not able to carry an exposimeter, can be predicted from their behaviour using measurements of the microenvironments which are relevant for very small children.

We regard our suggestions as basic requirements for future studies. Of course, additional features may be added to this core protocol. For instance, personal measurements of extremely low frequency magnetic fields may be added to the measurement study as has been done in the Netherlands [24]. Another possibility could be to compare geo-referenced personal measurements with the results from propagation models of fixed site transmitters [26] or with spot measurements or to evaluate changes in the exposure situation over a period of a few years.

The conduct of personal measurements is important for several reasons. In the past, mobile phones were a very important source of RF-EMF exposure mainly to the head for everybody who used them [1]. As a consequence most of the human experimental and epidemiological studies focused on mobile phone exposure and did not need personal exposure measurements. However, for future research, a change in exposure patterns can be expected. Firstly, the average output power of new UMTS phones is considerably lower than of GSM phones [33]. Secondly, there is an increasing number of new technologies such as Wireless Local Area Network (W-LAN), Worldwide Interoperability for Microwave Access (WiMax), Radio Frequency Identification (RFID) or Near Field Communication (NFC), contributing to an individual's exposure. Exposure of the general population to these sources is complex and concerns the whole body. Thus, personal exposimeter measurements are useful to better characterize multi-source exposure in the everyday environment.

In principle, one could also use exposimeters in epidemiological studies in order to directly measure individual exposure. However, this approach has several limitations: it is very costly and time-consuming for large studies, and long term measurements are not feasible and need considerable commitment of the study participants which results in a decreased participation rate. Participants might even manipulate the measurements by placing the exposimeter at positions where high RF-EMF exposures are expected. This makes exposimeters unattractive for direct exposure measurements in many epidemiological applications and well-designed personal exposure measurement studies are needed to increase our knowledge about the exposure distribution in the population and its relevant contributors. This facilitates the interpretation of previous RF-EMF research and helps to develop reliable exposure prediction models [34] for future studies. Such reliable exposure assessment methods are urgently needed to conduct epidemiological studies on potential health effects of long-term low dose exposure to RF-EMF in our everyday environment. Although the public is concerned about health risks from this type of exposure, methodologically sound studies are scarce and published studies do not allow firm conclusions to be drawn [35].

Knowledge of the exposure distribution is also needed for health risk assessment and risk communication. In this context it is crucial that study results are representative and comparable, and that exposure differences reflect real differences and are not due to methodological differences. Comparability of exposure measurements is also important for evaluating different approaches to reduce exposure, including environmental measures (e.g. reduced standard limits) and behavioural changes. For instance it will be interesting to evaluate whether exposure from mobile phone base stations in countries with lower standard limits (e.g. Switzerland, Italy) differs from that in the rest of Europe. Differences in standard limits might lead to a different architecture of mobile communication networks, e.g. a higher number of base stations with lower power, leading consequently to different emission patterns.

Exposimeters facilitate the collection of comprehensive data on personal exposure. Personal exposure to various RF-EMF sources can be assessed separately and different types of exposure metrics can be calculated, such as time spent above a certain threshold, rate of change, or other measures reflecting the intrinsic structure of the exposure data, as presented in Table 3. This is important because no biological mechanisms in the low dose range are known yet, hence, it remains unclear which aspects of exposure are relevant for health, if any at all. It has been speculated that effects may be frequency or modulation dependent [36] and, in such a case, estimating average exposure would not be the most appropriate exposure metric.

Exposimeters also have limitations, including the lack of measurement of all sources in the RF-EMF spectrum. At a population level, omitting data on RF-EMF from such sources is not expected to be important, however in specific situations such sources can be relevant, for instance, if someone lives close to a short wave transmitter. Shielding of the body, when carrying the exposimeter, is also a problem. A recent study has estimated that on average the electric field of different frequency bands is underestimated by as much as 64% [27]. In principle, factors could be used to correct the measurements. However, too few investigations in different microenvironments have been made so far for us to feel comfortable in proposing the application of such correction factors at the moment.

The most important limitation of the exposimeter concerns measurements of exposure from mobile phone handsets and other sources that are operated close to the body. In this case the measurement depends on the distance between the emitting device and the exposimeter rather than the distance between the device and the body. Hence, the measurement does not accurately reflect exposure of the body. This could be taken into account by estimating the whole-body SAR_wb _(Specific Absorption Rate) for each source by taking into account the average field distributions and field propagation for different typical exposure situations and microenvironments as proposed in [25,37]. In doing so, the same measured electric field strength for two frequency bands could mean different SAR_wb _depending on the typical usage/exposure situation for the corresponding source (e.g. near field from mobile phone vs. far field from mobile phone base station). In this way the exposimeter would extend to a "SAR_wb_-meter" and one could make an analysis in combination with both E-fields and actual whole-body SAR values, enabling future studies to make a comparison of personal exposure with basic restrictions [38]. This is a promising approach and its feasibility should be investigated in future studies.

## Conclusions

In this paper, experiences of various investigators with personal RF-EMF measurement studies are summarized. Based on these experiences criteria for future studies have been developed. Applying such common core procedures in future personal measurement studies is necessary so that observed differences in measurement studies reflect real exposure differences and not merely differences in the methods used.

## List of abbreviations

dB: Decibel; DECT: Digital Enhanced Cordless Telecommunications; EMF: Electromagnetic Field; FM: Frequency Modulated (radio broadcasting); GPS: Geographic Position System; GSM: Global System for Mobile Communications; NFC: Near Field Communication; RF-EMF: Radiofrequency electromagnetic field; RFID: Radio Frequency Identification; ROS: Robust regression on order statistics; SAR_wb_: Whole-body specific absorption rate; UMTS: Universal Mobile Telecommunications System; W-LAN: Wireless Local Area Network; WiMax: Worldwide Interoperability for Microwave Access.

## Competing interests

The authors declare that they have no competing interests.

## Authors' contributions

All authors contributed to the methodological discussions. PF conducted the statistical analyses. MR drafted the manuscript with input from all authors. RCP and SM have proofread the manuscript. All authors read and approved the final manuscript.
